# Optimization of spin Hall magnetoresistance in heavy-metal/ferromagnetic-metal bilayers

**DOI:** 10.1038/s41598-020-67450-3

**Published:** 2020-07-01

**Authors:** Łukasz Karwacki, Krzysztof Grochot, Stanisław Łazarski, Witold Skowroński, Jarosław Kanak, Wiesław Powroźnik, Józef Barnaś, Feliks Stobiecki, Tomasz Stobiecki

**Affiliations:** 1 Institute of Molecular Physics, Polish Academy of Sciences, ul. M. Smoluchowskiego 17, 60-179 Poznań, Poland; 2 Department of Electronics, AGH University of Science and Technology, Al. Mickiewicza 30, 30-059 Kraków, Poland; 3 Faculty of Physics and Applied Computer Science, AGH University of Science and Technology, al. Mickiewicza 30, 30-059 Kraków, Poland; 4 Faculty of Physics, Adam Mickiewicz University, ul. Uniwersytetu Poznańskiego 2, 61-614 Poznań, Poland

**Keywords:** Magnetic devices, Electronic devices, Magnetic properties and materials, Spintronics

## Abstract

We present experimental data and their theoretical description on spin Hall magnetoresistance (SMR) in bilayers consisting of a heavy metal (H) coupled to in-plane magnetized ferromagnetic metal (F), and determine contributions to the magnetoresistance due to SMR and anisotropic magnetoresistance (AMR) in five different bilayer systems: $\hbox {W}/\text {Co}_{20}\text {Fe}_{60}\text {B}_{20}$, $\text {Co}_{20}\text {Fe}_{60}\text {B}_{20}/\hbox {Pt}$, $\hbox {Au}/\text {Co}_{20}\text {Fe}_{60}\text {B}_{20}$, W/Co, and Co/Pt. The devices used for experiments have different interfacial properties due to either amorphous or crystalline structures of constitutent layers. To determine magnetoresistance contributions and to allow for optimization, the AMR is explicitly included in the diffusion transport equations in the ferromagnets. The results allow determination of different contributions to the magnetoresistance, which can play an important role in optimizing prospective magnetic stray field sensors. They also may be useful in the determination of spin transport properties of metallic magnetic heterostructures in other experiments based on magnetoresistance measurements.

## Introduction

Spin Hall magnetoresistance (SMR) is a phenomenon that consists in resistance dependence on the relative orientation of magnetization and spin accumulation at the interface of ferromagnet and strong spin-orbit material (such as 5*d* metals^1–8^, topological insulators^9^, or some 2D systems ^10^). In transition metals such as W and Pt, the spin accumulation results from spin current driven by the spin Hall effect (SHE)^11–14^. The spin current diffuses then into the ferromagnet or exerts a torque on the magnetization while being backscattered. Due to the inverse spin Hall effect (ISHE), the backscattered spin current is converted into a charge current that flows parallel to the bare charge current driven by external electric field, which effectively reduces the resistance^3,4^. One of the most important advantages of driving spin currents by SHE is that the spin currents can be induced by a charge current flowing in the plane of the sample^15^. This may remedy some obstacles on the road to further miniaturization of prospective electronic components, which have been encountered in spin-valves and magnetic tunnel junctions when the electric field is applied perpendicularly to interfaces. One of the drawbacks, however, is that the strength and effectiveness of such subtle effects depend strongly on the quality and spin properties of interfaces^16–23^.

Although early SMR experiments were performed on heavy-metal/ferromagnetic-insulator bilayers^1^, recent efforts are focused on the bilayers with ferromagnetic metallic layers, such as Co or $\text {Co}_{20}\text {Fe}_{60}\text {B}_{20}$ ones^5,7^, which are currently more relevant for applications. When the magnetization is parallel to the spin accumulation, the spin current from the heavy-metal can easily diffuse into the ferromagnetic metal (influencing its spin transport properties and spin accumulation on the ferromagnetic metal side)^[5,24–30^. This is especially important when an additional spin sink (another heavy-metal layer or an antiferromagnet) is on the other side of the ferromagnetic layer, where effects such as spin current interference might take place^6^.

Moreover, as charge current flows in plane of the sample, additional phenomena may occur, such as anisotropic magnetoresistance (AMR) or anomalous Hall effect (AHE)^31–36^. These effects can obscure determination of spin transport parameters and make evaluation of the SMR contribution to the measured magnetoresistance more difficult. Since the determination of such transport properties as the spin Hall angle (which parameterizes strength of the spin Hall effect) and spin diffusion length in different experimental schemes, for instance in spin-orbit torque ferromagnetic resonance (SOT-FMR)^37,38^, relies heavily on the magnetoresistive properties of a system, it is important to properly determine all the contributions to magnetoresistance.

Here, we revisit the theory of spin Hall magnetoresistance in metallic bilayers by explicitly including the contribution from AMR into the spin drift-diffusion theory for the ferromagnetic metal layer. The expressions for magnetoresistance are then fitted to the data obtained from resistance measurements on heavy-metal (H)/ferromagnet (F) bilayers, where H: W, Pt, Au, while F: Co, $\text {Co}_{20}\text {Fe}_{60}\text {B}_{20}$. This allows us to determine more accurately contributions from various magnetotransport phenomena occurring in metallic bilayers where the spin Hall effect is the driving source. Such analysis may also be useful in the efforts to optimize prospective devices for information technology.

## Results

**Table 1:** Composition of samples, and resistivities of heavy metal and ferromagnetic layers. Numbers in parentheses next to material symbol denote thickness (in nm) of the corresponding layer; parameters used for fitting the model to experimental data.

No.	Sample	$\rho _0^{\text {H}}$ ($\mu \Omega \text {cm}$)	$\rho _0^{\text {F}}$ ($\mu \Omega \text {cm}$)	$|\theta _{\mathrm{SH}}|$ (%)	$\theta _{\mathrm{AMR}}$ (%)	$\lambda _{\mathrm{H}}$ (nm)	$\lambda _{\mathrm{F}}$ (nm)
W1	$\hbox {W}(5)/\text {Co}_{20}\text {Fe}_{60}\text {B}_{20}(t_F)/\hbox {Ta}(1)$	185	144	$22.5\pm 0.4$	$0.19\pm 0.03$	$1.3^a$	$5^c$
W2	$\hbox {W}(t_H)/\text {Co}_{20}\text {Fe}_{60}\text {B}_{20}(5)/\hbox {Ta(1)}$	166	144	$21\pm 1$	$0.07\pm 0.08$	$1.3^a$	$5^c$
W3	$\hbox {W}(5)/\hbox {Co}(t_F)/\hbox {Ta}(1)$	120	22	$\approx 0$	$1.1\pm 0.3$	$1.3^a$	$7^c$
W4	$\hbox {W}(t_H)/\hbox {Co}(5)/\hbox {Ta}(1)$	120	30	$23\pm 2$	$0.69\pm 0.02$	$1.3^a$	$7^c$
P1	$\text {Co}_{20}\text {Fe}_{60}\text {B}_{20}(t_F)/\hbox {Pt}(3)$	95	102	$16.0\pm 0.2$	$0.38\pm 0.01$	$2.2^a$	$5^c$
P2	$\text {Co}_{20}\text {Fe}_{60}\text {B}_{20}(5)/\hbox {Pt}(t_H)$	151	161	$6\pm 2$	$0.36\pm 0.03$	$2.2^a$	$5^c$
P3	$\hbox {Co}(t_F)/\hbox {Pt}(4)$	55	18	$\approx 0$	$0.5\pm 0.1$	$2.2^a$	$7^b$
P4	$\hbox {Co}(5)/\hbox {Pt}(t_H)$	24	57	$9\pm 1$	$1.5\pm 0.1$	$2.2^a$	$7^b$
A1	$\hbox {Ti}(2)/\hbox {Au}(5)/\text {Co}_{20}\text {Fe}_{60}\text {B}_{20}(t_F)/\hbox {Ti}(1.5)$	24	96	$3.3\pm 0.1$	$0.210\pm 0.008$	$1.6^a$	$5^c$
A2	$\hbox {Ti}(2)/\hbox {Au}(t_H)/\hbox {CoFeB}(5)/\hbox {Ti}(1.5)$	15	96	$\hbox {5}\pm 1$	$0.33\pm 0.05$	$1.6^a$	$5^c$

$^a$Ref. 38, $^b$Ref. 39, $^c$Ref. 40.

This section is divided into two sections depending on the ferromagnetic material used in the bilayer. As $\text {Co}_{20}\text {Fe}_{60}\text {B}_{20}$ is amorphous and Co is crystalline, they present differently in magnetoresistance experiments and can influence the estimation of spin transport properties, especially when the thickness of ferromagnetic material is varied while thickness of heavy metal remains constant.

The spin Hall angles obtained from fitting for W, Pt, and Au, shown in Table 1, agree quite well with spin Hall angles for thick heavy metals obtained in spin-orbit torque ferromagnetic resonance experiments used in our previous papers^38,41^. Larger spin Hall angle for Pt-based bilayer P1, where the thickness of ferromagnetic metal varies, can be attributed to, f.i., changes in spin-mixing conductivity^17^, which, as described above, we do not take into account in current approach.

### $\text {Co}_{20}\text {Fe}_{60}\text {B}_{20}$-based bilayers

**Fig. 1:**
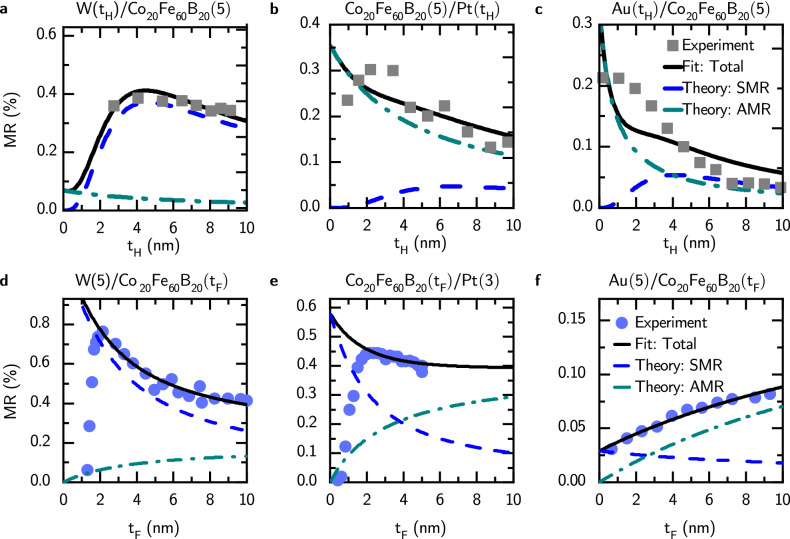
Relative magnetoresistance, *MR*, as a function of heavy metal’s thickness, $t_{\mathrm{H}}$, for: **a**, $\hbox {W}/\text {Co}_{20}\text {Fe}_{60}\text {B}_{20}$, **b**, $\text {Co}_{20}\text {Fe}_{60}\text {B}_{20}/\hbox {Pt}$, and **c**, $\hbox {Au}/\text {Co}_{20}\text {Fe}_{60}\text {B}_{20}$ bilayers, and as a function of ferromagnetic metal’s thickness, $t_{\mathrm{F}}$, for: **d**, $\hbox {W}(5)/\text {Co}_{20}\text {Fe}_{60}\text {B}_{20}$, **e**, $\text {Co}_{20}\text {Fe}_{60}\text {B}_{20}/\hbox {Pt}(3)$, and **f**, $\hbox {Au}(5)/\text {Co}_{20}\text {Fe}_{60}\text {B}_{20}$ bilayers. Parameters used for theoretical curves are gathered in Table 1.

Figure 1 shows relative magnetoresistance as a function of heavy metal (Fig. 1a–c) and ferromagnetic metal (Fig. 1d–f) layer thicknesses for $\hbox {H}/\text {Co}_{20}\text {Fe}_{60}\text {B}_{20}$ bilayers where H: W, Pt, Au.

Dependence of magnetoresistance on heavy-metal thickness, with fixed $t_{\mathrm{F}}=5~\text {nm}$, shown in Fig. 1a–c indcates, as expected, that SMR is the largest contribution to magnetoresistance in heterostructure with W as a heavy metal layer due to larger spin Hall angle of W, $|\theta _{\mathrm{SH}}|\approx 21\%$, compared to Pt, $|\theta _{\mathrm{SH}}|\approx 6\%$, and to Au, $|\theta _{\mathrm{SH}}|\approx 4\%$. Consequently, in Pt and Au bilayers AMR dominates over SMR.

The dependence of magnetoresistance on ferromagnetic layer thickness is shown in Fig. 1d–f. For 5 nm–thick W as heavy metal layer, shown in Fig. 1d SMR is still the dominating contribution to the total magnetoresistance in the studied thickness range. For device with 3 nm–thick Pt, the SMR for $t_{\mathrm{F}}\gtrsim 2~\text {nm}$ is smaller than AMR. Note that for both W- and Pt-based bilayers the model fit and theoretical prediction do not describe the behavior of MR for $t_{\mathrm{F}}\lesssim 2~\text {nm}$, which can be attributed to strong dependence of the interfacial parameters such as spin-mixing conductance on thickness. Due to small spin Hall angle, SMR in Au-based is rather small and MR is dominated by AMR.

### Co-based bilayers

**Fig. 2:**
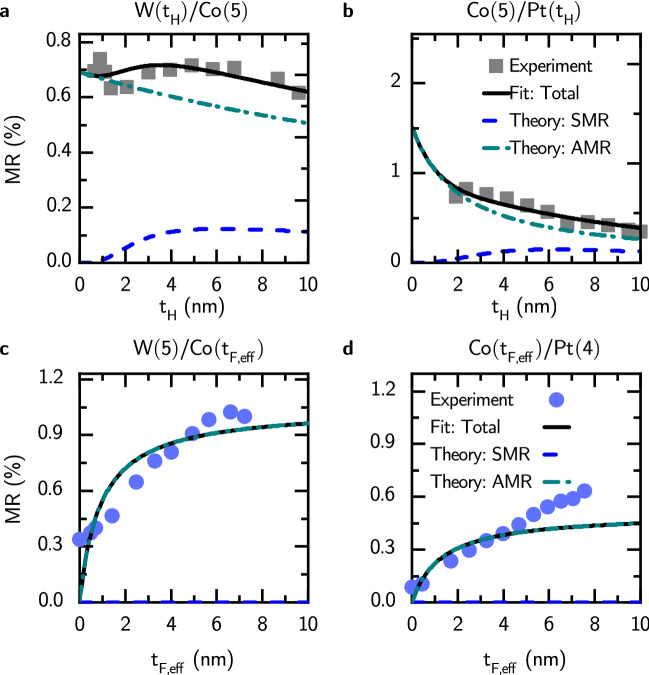
Relative magnetoresistance, *MR*, as a function of heavy metal’s thickness, $t_{\mathrm{H}}$, for: **a**, $\hbox {W}(t_{\mathrm{H}})/\hbox {Co}(5)$, **b**, $\hbox {Co}(5)/\hbox {Pt}(t_{\mathrm{H}})$ bilayers, and as a function of ferromagnetic metal’s effective thickness, $t_{\mathrm{F},\text {eff}}$, for: **c**, $\hbox {W}(5)/\hbox {Co}(t_{\mathrm{F},\text {eff}})$, **d**, $\hbox {Co}(t_{\mathrm{F},\text {eff}})/\hbox {Pt}(4)$ bilayers. Parameters used for theoretical curves are gathered in Table 1.

Magnetoresistance and relative magnetoresistance in H/Co bilayers (H: W, Pt) are shown in Fig. 2 as a function of heavy metal (Fig. 2a,b) and ferromagnetic metal (Fig. 2c,d) layer thicknesses.

For W and Pt bilayers with varying heavy metal thickness and $t_{\mathrm{F}}=5~\text {nm}$, shown in Fig. 2, the total magnetoresistance is mostly due to AMR, in contrast to $\text {Co}_{20}\text {Fe}_{60}\text {B}_{20}$–based bilayers described in the previous subsection.

For bilayers with varying ferromagnetic metal (Co) thickness, shown in Fig. 2c,d, the total magnetoresistance is also largely dominated by AMR.

Due to existence of a magnetic dead layer, disoriented crystalline structure of Co, and due to the fact that magnetization of Co does not lie completely in-plane of the sample for small thicknesses we introduced the magnetically effective thickness, $t_{\mathrm{F},\text {eff}}$, of Co layer. More details on some of these aspects can be found in Supplementary Information. Since the thickness of heavy-metal is fixed and the thickness of the ferromagnetic metal increases, the large differences in resistivities result in larger portion of charge current flowing into Co leading to negligible SMR—thus preventing proper estimation of spin Hall angle of both W and Pt.

## Discussion

Our systematic analysis of magnetoresistance in in-plane magnetized heavy-metal/ferromagnetic-metal bilayers with crystalline Co and amorphous $\text {Co}_{20}\text {Fe}_{60}\text {B}_{20}$ has shown that proper choice of ferromagnetic-metal is crucial to the optimization of spin Hall magnetoresistance.

As shown in previous section, although W has larger spin Hall angle than Pt and Au, the magnetoresistance of W/$\text {Co}_{20}\text {Fe}_{60}\text {B}_{20}$ and even W/Co (for thin W) bilayers can be lower than that of Co/Pt bilayer due to high AMR contribution in the latter. This also leads to possible underestimation of SMR contribution which is lower in W/Co than in W/$\text {Co}_{20}\text {Fe}_{60}\text {B}_{20}$ bilayers, one of the main reasons for which is quite large difference in resistivities of both layers (see Table 1), due to the fact that here $\beta$-W phase is disoriented (amorphous-like) resulting in more current flowing through Co than W and on average smaller spin Hall effect (see Supplementary Information). Moreover, due to the fact that here W is mostly amorphous and Co crystalline, a different interface properties between these materials than between crystalline-crystalline or amorphous-amorphous bilayers can influence spin transport as well.

For materials with stronger spin-orbit coupling (W and Pt) and comparable resistivities to $\text {Co}_{20}\text {Fe}_{60}\text {B}_{20}$, one obtains higher magnetoresistance response with thinner ferromagnet. In the case of Au-based bilayer, whose resistivity is smaller than that of $\text {Co}_{20}\text {Fe}_{60}\text {B}_{20}$, one obtains higher magnetoresistance with thicker ferromagnet. The predicted SMR contribution for $\hbox {Au}/\text {Co}_{20}\text {Fe}_{60}\text {B}_{20}$ can be higher than the SMR contribution for $\text {Co}_{20}\text {Fe}_{60}\text {B}_{20}$/Pt due to the fact that larger current density flows through Au than through Pt, thus increasing the spin Hall response.

The estimation of SMR in the case of metallic bilayers is hindered by large differences in resistivity of the constituent metallic layers. Since in this case AMR is strongly dominating, the total MR increases with increasing effective thickness of Co.

In conclusion, we have developed an extended model of magnetoresistance for magnetic metallic bilayers with in-plane magnetized ferromagnets, which explicitly includes SMR and AMR contributions. The model was then fitted to experimental data on magnetoresistance in: $\hbox {W}/\text {Co}_{20}\text {Fe}_{60}\text {B}_{20}$, $\text {Co}_{20}\text {Fe}_{60}\text {B}_{20}/\hbox {Pt}$, $\hbox {Au}/\text {Co}_{20}\text {Fe}_{60}\text {B}_{20}$, W/Co, Co/Pt heterostructures, to estimate the strength of SMR and AMR effects. In particular, we have compared the influence of amorphous ferromagnet ($\text {Co}_{20}\text {Fe}_{60}\text {B}_{20}$) and crystalline ferromagnet (Co) on total magnetoresistance and analyzed the dependence of magnetoresistance on ferromagnet’s thickness, which allows for better optimization of magnetic bilayers.

These results allow for a more accurate estimation of different contributions to magnetoresistance in magnetic metallic systems, which is important for applications in, e. g., spintronic SOT-devices^42^ or in other experimental schemes that rely on magnetoresistance measurements in evaluation of the spin transport properties.

## Methods

### Experiment

Table 1 shows the multilayer systems that were produced for SMR studies. The magnetron sputtering technique was used to deposit multilayers on the $\hbox {Si/SiO}_2$ thermally oxidized substrates. Thickness of wedged layers were precisely calibrated by X-ray reflectivity (XRR) measurements. The details of sputtering deposition parameters as well as structural phase analysis of highly resistive W and Pt layers can be found in our recent papers^38,41^. Au in $\hbox {Au}/\text {Co}_{20}\text {Fe}_{60}\text {B}_{20}$ bilayers is (111) fcc textured similarly as Pt in $\text {Co}_{20}\text {Fe}_{60}\text {B}_{20}/\hbox {Pt}$ bilayer^38^. In turn, structure analysis of the hcp-Co crystal phases grown on disoriented $\beta$-W can be found in the Supplementary Information.

After deposition, multilayered systems were nanostructured using either electron-beam lithography or optical lithography, ion etching and lift-off. The result was a matrix of Hall bars and strip nanodevices for further electrical measurements. The sizes of produced structures were: 100 $\mu \hbox {m}$ x 10 $\mu \hbox {m}$ or 100 $\mu \hbox {m}$ x 20 $\mu \hbox {m}$. In order to ensure good electrical contact with the Hall bars and strips, Al(20)/Au(30) contact pads with dimensions of 100 $\mu \hbox {m}$ x 100 $\mu \hbox {m}$ were deposited. Appropriate placement of the pads allows rotation of the investigated sample and its examination at any angle with respect to the external magnetic field in a dedicated rotating probe station using a four-points probe. The constant magnetic field, controlled by a gaussmeter exceeded magnetization saturation in plane of the sample and the sample was rotated in an azimuthal plane from $-120^{\circ }$ to $+100^{\circ }$.

The resistance of the system was measured with a two- and four-point technique using Keithley 2400 sourcemeters and Agilent 34401A multimeter. As shown in Supplementary Information, resistances of bilayers with amorphous ferromagnet $\text {Co}_{20}\text {Fe}_{60}\text {B}_{20}$ are about one order higher than these with polycrystalline Co. The same results were obtained using both methods. The thickness-dependent resistivity of individual layers was determined by method described in Ref. 7, and by a parallel resistors model. For more details on resistivity measurements we refer the reader to Supplementary Information.

### Theory

To properly assess all contributions to magnetoresistance one should find first the average current density flowing through the whole heterostructure. This approach, in contrast to the one described in Ref. 5 allows one to properly describe magnetoresistance in more complicated heterostructures, where *ad hoc* addition of consitutent terms might lead to oversimplification and improper determination of different components in the magnetoresistance. Moreover, calculating average current density allows for a phenomenological description of how various magnetoresistance effects depend on thicknesses of the constituent layers. The drawback, however, is the necessary simplification of fitting parameters, which we discuss in more detail in the next subsection devoted to fitting procedure.

Only the component flowing along the normal to interfaces is relevant and will be taken into account in the following, i.e.

1 $$\begin{aligned} \mathbf {j}_s^{\text {H}}(z)=-\frac{\theta _{\mathrm{SH}}}{\rho _0^{\text {H}}}\hat{\mathbf {e}}_z\times \mathbf {E}+\frac{1}{2e\rho _0^{\text {H}}}\frac{\partial \varvec{\mu }_s^{\text {H}}(z)}{\partial z}\,. \end{aligned}$$

Here $\theta _{\mathrm{SH}}$ is the spin Hall angle, $\rho _0^{\text {H}}$ is the bare resistivity of the heavy metal, and $\varvec{\mu }_s^{\text {H}}(z)$ is the spin accumulation that is generally *z*-dependent.

The charge current density in the heavy-metal (H) layer, in turn, can be written in the form

2 $$\begin{aligned} \mathbf {j}_c^{\text {H}}(z)=\frac{1}{\rho _0^{\text {H}}}\mathbf {E}+\frac{\theta _{\mathrm{SH}}}{2e\rho _0^{\text {H}}}\hat{\mathbf {e}}_z\times \frac{\partial \varvec{\mu }_s^{\text {H}}(z)}{\partial z}\,, \end{aligned}$$

and contains the bare charge current density and the current due to inverse spin Hall effect. Note, that the spin current in general can induce charge current also flowing along the axes *x* and *y*. However, due to lateral dimensions of the samples much larger than the layer thicknesses and spin diffusion lengths, those additional components can be neglected.

Thus, one can write^28,29^:

3 $$\begin{aligned} \mathbf {j}_s^{F}(z)&= \frac{1}{2e\rho _0^{\text {F}}}\nabla \mu _s^{\text {F}}(z) +\frac{\beta }{2e\rho _0^{\text {F}}}\nabla \mu _c^{\text {F}}(\mathbf {r}) -\frac{\theta _{\mathrm{AMR}}}{2e\rho _0^{\text {F}}}\hat{\mathbf {m}} \left[ \hat{\mathbf {m}}\cdot \nabla \mu _s^{\text {F}}(z) \right] \,, \end{aligned}$$

in which $\theta _{\mathrm{AMR}}$ is the AMR angle, defined as $\theta _{\mathrm{AMR}}=\sigma _{\mathrm{AMR}}\rho _0^{\text {F}}$, while ${\mu _c^{\text {F}}(\mathbf {r})=2e\mathbf {E}\cdot \mathbf {r}+\mu _c^{F}(z)}$ is the electrochemical potential.

Charge current density in the ferromagnetic layer (F) can be written as^28,29^,

4 $$\begin{aligned} \mathbf {j}_c^{F}(z)&=\frac{1}{2e\rho _0^{\text {F}}}\nabla \mu _c^{\text {F}}(\mathbf {r})+\frac{\beta }{2e\rho _0^{\text {F}}} \nabla \mu _s^{\text {F}}(z)-\frac{\theta _{\mathrm{AMR}}}{2e\rho _0^{\text {F}}}\hat{\mathbf {m}}\left[ \hat{\mathbf {m}} \cdot \nabla \mu _c^{\text {F}}(\mathbf {r}) \right] . \end{aligned}$$

Note, in the above equations the current densities in both H and F layers we assumed as linear response to electric field, i.e. we neglected the so-called unidirectional spin Hall magnetoresistance effect^24–27^.

The spin current $\mathbf {j}_s^{\text {HF}}$ flowing through the heavy-metal/ferromagnet interface is given by the following expression [43]:

5 $$\begin{aligned} \mathbf {j}_s^{\text {HF}}&=G_F\bigg [\left( \varvec{\mu }_s^{\text {F}}(0) -\varvec{\mu }_s^{\text {H}}(0)\right) \cdot \hat{\mathbf {m}} \bigg ]\hat{\mathbf {m}}+G_r\hat{\mathbf {m}}\times \hat{\mathbf {m}} \times \varvec{\mu }_s^{\text {H}}(0)\,. \end{aligned}$$

Here $G_F=(1-\gamma ^2)(G_{\uparrow }+G_{\downarrow })/2$ with $\gamma$ defined as $\gamma =(G_\uparrow -G_\downarrow )/(G_{\uparrow }+G_{\downarrow })$ and $G_\uparrow$ and $G_\downarrow$ denoting the interface conductance for spin-$\uparrow$ and spin-$\downarrow$. Furthermore, $G_{r}\equiv {\text {Re}}G_{\mathrm{mix}}$ and $G_{i}\equiv {\text {Im}}G_{\mathrm{mix}}$, where $G_{\mathrm{mix}}$ is the so-called spin-mixing conductance. Note, that we neglect explicitly a contribution from the interfacial Rashba-Edelstein spin polarization^38^. A strong interfacial spin-orbit contribution which induces spin-flip processes can also be combined with the interfacial spin conductance $G_F$ as a spin-conductance reducing parameter $1-\eta$, with $\eta =0$ for no interfacial spin-orbit coupling, and $\eta =1$ for maximal spin-orbit coupling. Note, that this reduction could also be attributed to the magnetic proximity effect, especially in the case of Pt-based heterostructures^13^, however recent studies suggest its irrelevance for spin-orbit-torque–related experiments^22^. In the following discussion we assume $\eta =0$ and treat $G_F$ as an effective parameter.

To find charge and spin currents we need to find first the spin accumulation at the H/F interface and also at external surface/interfaces. This can be found from the following boundary conditions:

6a $$\begin{aligned} \mathbf {j}_s^{\text {H}}(z=-t_H)=0 \,, \end{aligned}$$

6b $$\begin{aligned} \mathbf {j}_s^{\text {F}}(z=t_F)=0 \,, \end{aligned}$$

6c $$\begin{aligned} \mathbf {j}_s^{\text {H}}(z=0)=\mathbf {j}_s^{\text {HF}}\,, \end{aligned}$$

6d $$\begin{aligned} j_{s,z}^{\text {F}}(z=0)=\mathbf {j}_s^{\text {HF}}\cdot \hat{\mathbf {m}}\,. \end{aligned}$$

Having found electrochemical potential and spin accumulation from general solution

7 $$\begin{aligned} \mathbf {\mu }_s^{F,H}(z)=\mathbf {A}_{F,H} e^{-z/\lambda _{F,H}}+\mathbf {B}_{F,H} e^{z/\lambda _{F,H}}\,, \end{aligned}$$

where $\mathbf {A}_{F,H}$ and $\mathbf {B}_{F,H}$ are coefficients to be determined and $\lambda _{F,H}$ is the spin diffusion length in ferromagnet or heavy metal, one can find the longitudinal in-plane components of the averaged charge current $\mathbf {j}(\hat{\mathbf {m}})$ from the formula:

8 $$\begin{aligned} j_{xx}(\hat{\mathbf {m}})=\frac{1}{t_H+t_F}\left[ \int _{t_H}dz \hat{\mathbf {e}}_{x}\cdot \mathbf {j}_c^{\text {H}}(z)+\int _{t_F}dz \hat{\mathbf {e}}_{x}\cdot \mathbf {j}_c^{\text {F}}(z)\right] \,. \end{aligned}$$

The total longitudinal charge current can be written down in the Ohm’s-law form,

9 $$\begin{aligned} j_{xx}(\hat{\mathbf {m}})=\frac{1}{\rho _{xx}(\hat{\mathbf {m}})}E_x\,, \end{aligned}$$

where the longitudinal resistivity is defined as follows:

10 $$\begin{aligned} \frac{1}{\rho _{xx}(\hat{\mathbf {m}})}&=\sigma _0+\sigma _xm_x^2+\sigma _ym_y^2 \end{aligned}$$

with

11 $$\begin{aligned} \sigma _0&\approx \frac{\rho _0^{\text {F}} t_H+\rho _0^{\text {H}} t_F}{\rho _0^{\text {F}} \rho _0^{\text {H}} t_F+\rho _0^{\text {F}} \rho _0^{\text {H}} t_H} \end{aligned}$$

12 $$\begin{aligned} \sigma _x&=\frac{\theta _{\mathrm{SH}}^2}{\rho _0^{\text {H}}}\frac{g_{\mathrm{DL}}\lambda _{\mathrm{H}} }{t_F+t_H}\tanh \left( \frac{t_H}{2 \lambda _{\mathrm{H}}}\right) -\frac{\theta _{\mathrm{AMR}} }{\rho _0^{\text {F}}}\frac{t_F}{t_F+t_H}\,, \nonumber \\&=\sigma _x^{\text {SH}}+\sigma _x^{\text {AMR}}\,, \end{aligned}$$

13 $$\begin{aligned} \sigma _y&=\frac{\theta _{\mathrm{SH}}^2}{\rho _0^{\text {H}}}\frac{ g_H^{\text {F}}\lambda _{\mathrm{H}}}{t_F+t_H}\tanh \left( \frac{t_H}{2 \lambda _{\mathrm{H}}}\right) \,. \end{aligned}$$

In the above expressions the following dimensionless coefficients have been introduced to simplify the notation:

14 $$\begin{aligned} g_{\mathrm{DL}}&=\left[ 1-{\text {sech}}{\left( \frac{t_H}{\lambda _{\mathrm{H}}}\right) }\right] \frac{g_r(1+g_r)+g_i^2}{(1+g_r)^2+g_i^2} \,, \end{aligned}$$

15 $$\begin{aligned} g_{r,i}&=2G_{r,i}\rho _0^{\text {H}}\lambda _{\mathrm{H}}\coth {\left( \frac{t_H}{\lambda _{\mathrm{H}}} \right) }\,, \end{aligned}$$

16 $$\begin{aligned} g_{H}^{\text {F}}&=\left[ 1-{\text {sech}}{\left( \frac{t_H}{\lambda _{\mathrm{H}}}\right) }\right] \frac{1}{1+\left( \frac{1}{2G_F\lambda _{\mathrm{H}} \rho _0^{\text {H}}}+\gamma _H^{\text {F}}\right) \tanh \left( \frac{t_H}{\lambda _{\mathrm{H}}}\right) }\,, \end{aligned}$$

17 $$\begin{aligned} \gamma _{H}^{\text {F}}&=\frac{\lambda _{\mathrm{F}} \rho _0^{\text {F}}}{\lambda _{\mathrm{H}} \rho _0^{\text {H}}\left( 1-\beta ^2\right) }\coth \left( \frac{t_F}{\lambda _{\mathrm{F}}}\right) \,. \end{aligned}$$

With the resistivity defined in Eq. (10) we can now define magnetoresistance,

18 $$\begin{aligned} MR=\frac{\rho _{xx}(\hat{\mathbf {m}}\parallel \hat{\mathbf {e}}_x)-\rho _{xx}(\hat{\mathbf {m}}\parallel \hat{\mathbf {e}}_y)}{\rho _{xx}(\hat{\mathbf {m}}\parallel \hat{\mathbf {e}}_x)}\,, \end{aligned}$$

Taking into account Eqs. (11)-(13), the above formula can be written as,

19 $$\begin{aligned} MR\approx \frac{\sigma _y-\sigma _x}{\sigma _0}\,. \end{aligned}$$

In order to compare the models with and without AMR, we define SMR as:

20 $$\begin{aligned} SMR=MR\Bigg |_{\theta _{\mathrm{AMR}}\rightarrow 0}=\frac{\sigma _y^{\text {SH}}-\sigma _x^{\text {SH}}}{\sigma _0}\,, \end{aligned}$$

which simplifies our model to that introduced by Kim et al. 5. We also define AMR coefficient

21 $$\begin{aligned} AMR=MR\Bigg |_{\theta _{\mathrm{SH}}\rightarrow 0}=-\frac{\sigma _x^{\text {AMR}}}{\sigma _0}\,. \end{aligned}$$

### Fitting procedure

In order to analyze the experimental data in light of our extended model, we fit Eq. (19) to the data on relative magnetoresistance. We have assumed some constant values according to literature and our previous works: spin polarization at Fermi level of both Co and $\text {Co}_{20}\text {Fe}_{60}\text {B}_{20}$ are taken as $\beta =0.3$. Note that this value can range in $\text {Co}_{20}\text {Fe}_{60}\text {B}_{20}$ from 0.1 to 0.6^40^ and can influence the fit of the model to the data. We have assumed spin diffusion lengths in Pt and W from our previous papers^38,41^ to be 2.2 nm and 1.3 nm, respectively. For $\text {Co}_{20}\text {Fe}_{60}\text {B}_{20}$ we assumed constant room-temperature value of $\lambda _F\approx 5~\text {nm}$ ^40^ and for Co $\lambda _F\approx 7~\text {nm}$^39^. Note, that we have assumed constant effective spin diffusion lengths for the constituent layers obtained from our previous analyses ^38,41^. In general, however, these parameters can depend on temperature or thickness of the layers^44,45^. This fact can lead to underestimation of spin diffusion lengths and overestimation of the spin Hall angles. One of the remedies might be to use effective thickness-dependent parameters^45^. However, such approaches are still mostly empirical and not based on proper theoretical grounding and as such have their own limitations.

Moreover we have assumed transparent contacts for spin transport, i.e. $G_F\rightarrow \infty$ and $G_r\rightarrow \infty$, and also assumed $G_i$ to be negligible. These assumptions are mostly valid for metallic interfaces. However, these parameters can also strongly depend on type of interface, i.e. they can differ in amorphous/crystalline (f.i. $\text {Co}_{20}\text {Fe}_{60}\text {B}_{20}/\hbox {Pt}$), crystalline/crystalline (Co/Pt), and amorphous-like/amorphous (f.i. $\hbox {W}/\text {Co}_{20}\text {Fe}_{60}\text {B}_{20}$) heterostructures.

We have assumed spin Hall angle $\theta _{\mathrm{SH}}$ and AMR coefficient $\theta _{\mathrm{AMR}}$ as fitting parameters and the results of fitting the model to the experimental data on magnetoresistance are gathered in Table 1. Morevoer, we have assumed anomalous Hall effect to be negligible in the in-plane magnetized systems considered in the paper. This effect might play an important role for ferromagnets with stronger spin-orbit coupling or ferromagnets tilted out of plane^28–30^.

## Supplementary information

Supplementary information 1
