# Supplementary material for: Optimization of spin Hall magnetoresistance in heavy-metal/ferromagnetic-metal bilayers
Source: Sci Rep. 2020 Jul 1;10:10767. doi: 10.1038/s41598-020-67450-3 (PMC7329912; doi:10.1038/s41598-020-67450-3)
Supplement: Supplementary file 1 — Supplementary information 1 [file 41598_2020_67450_MOESM1_ESM.pdf]

Supplementary Information for

Optimization of Spin Hall Magnetoresistance in Heavy-metal/Ferromagnetic-metal Bilayers

Łukasz Karwacki [1,2], Krzysztof Grochot [2,3], Stanisław Łazarski [2], Witold Skowroński [2], Jarosław Kanak [2], Wiesław Powroźnik [2], Józef Barnaś [4,1], Feliks Stobiecki [1] and Tomasz Stobiecki [2,3]

[1] Institute of Molecular Physics, Polish Academy of Sciences, ul. Smoluchowskiego 17, 60-179 Poznań, Poland

[2] AGH University of Science and Technology, Department of Electronics, Al. Mickiewicza 30, 30-059 Kraków, Poland

[3] Faculty of Physics and Applied Computer Science, AGH University of Science and Technology, 30-059 Kraków, Poland

[4] Faculty of Physics, Adam Mickiewicz University, ul. Uniwersytetu Poznańskiego 2, 61-614 Poznań, Poland

Structural characterization

The samples studied in this paper were deposited using magnetron-sputtering technique. The details on deposition parameters as well as structural phase analysis for W/CoFeB, CoFeB/Pt, and Co/Pt bilayers were described in our recent papers [S1, S2]. In these publications we have shown that the tungsten layer grows in the disoriented β-W phase, CoFeB is amorphous, while Co and Pt are strongly textured in direction fcc (111). However, the growth of Co on disoriented β-W underlayer is quite unexpected. The grazing incidence X-ray diffraction (GIXD) profiles at incidence angle $\omega=1^{o}$ (this method was used in order to minimized the influence of W underlayer on top Co layer) show very weak hcp-Co peaks (Fig. S1). In the sample W3 (Fig. S1 (a)) we measured overlapping by β-W (210) hcp-Co(100) and decreasing reflection intensity of hcp-Co (101) with decreasing of Co thickness. Weak diffraction non-textured polycrystalline hcp-Co is due to growing hcp-Co on strongly disoriented β-W underlayer. A diffraction peak of hcp-Co(101) also appears in sample W4 (Fig. S1 (b)). In addition, as a result from GIXD measurements below 4 nm, W layers grow in the amorphous-like structure (Fig. S1 (b)).

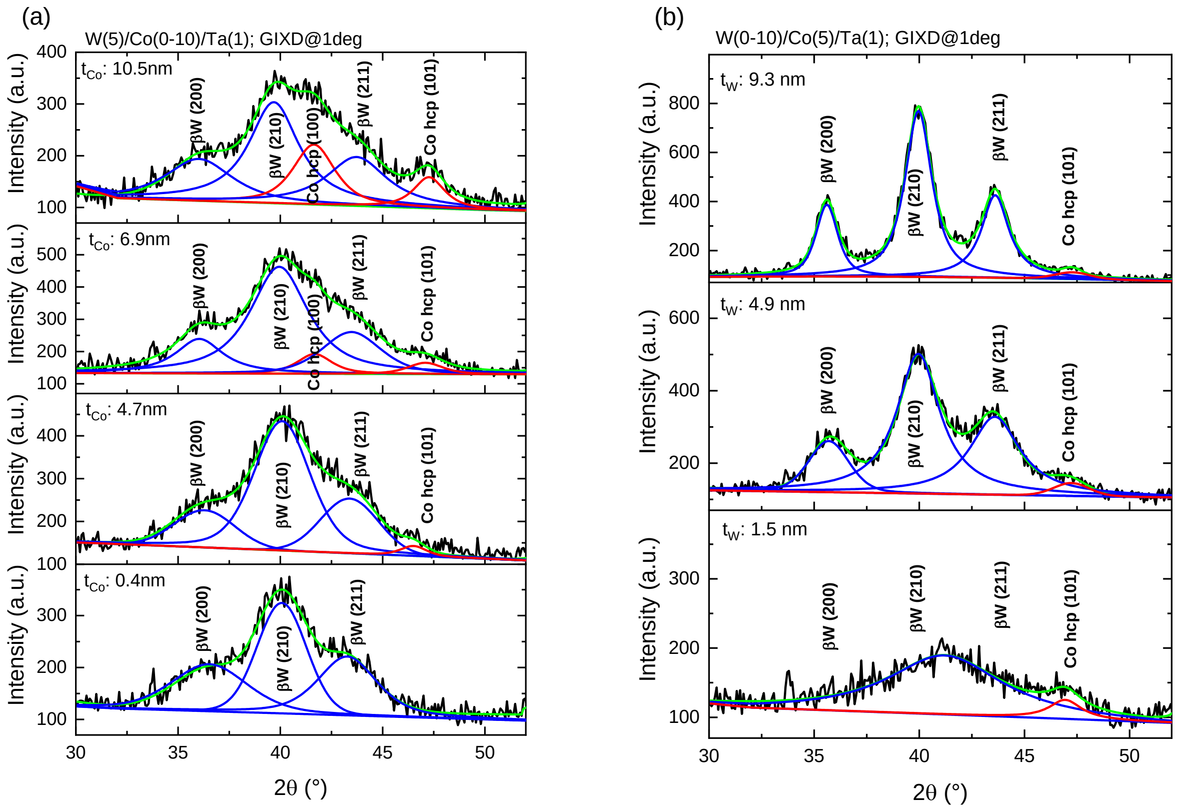

Fig. S1 GIXD profiles at incidence angle ω =1° : (a) sample W3: W(5)/Co(0-10)/Ta(1) (b) sample W4: W(0-10)/Co(5)/Ta(1).

Resistivity measurements

The resistivity of each material is determined from the measured sheet conductance G = L/(w·R) of each microstrip as a function of the thickness of heavy metals and ferromagnets according to the procedure described in Ref. [S3]. As shown in Fig. S2 sheet conductance of all investigated samples decreases linearly with thickness decreasing in the range of 10 nm to about 2 nm. For the majority of studied systems below 2 nm the deviation from linearity of G(t) dependence is due to transition from polycrystalline to amorphous-like structure. For example, below 2 nm, hcp-Co in the sample W3 and β-W in the sample W4 transformed to amorphous phase (Fig. S2(a)). Intersection of G(t) dependencies at 5 nm, for samples W3 and W4, shows complementary behavior in W/Co system. G(t) dependencies of the samples W1 and W2 are similar, due to high resistivities of β-W and amorphous CoFeB. However, sheet conductances for samples P1 and P2 with amorphous ferromagnet CoFeB are smaller than for samples P3 and P4 with polycrystalline fcc-Co (Fig. S2(b)). Table I contains resistivities for all studied systems obtained from sheet conductance measurements and calculated using parallel resistors model. To determine the resistivity in the W1, W2, W3 and W4 samples, the capping Ta layer was not taken into account because for a thickness of 2 nm Ta is oxidized and does not conduct. In the case of samples A1 and A2 Ti buffer and capping layers were also oxidized.

(a) (b)

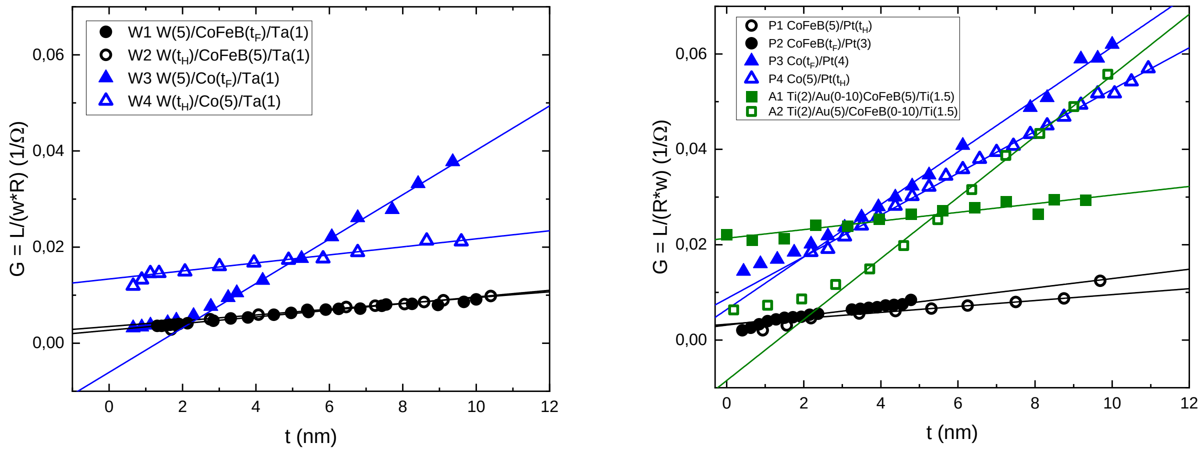

Fig. S2 Sheet conductance G as a function of heavy metal (W, Pt, Au) and ferromagnet (CoFeB, Co) thickness t for: (a) W/CoFeB and W/Co bilayers, (b) CoFeB/Pt, Co/Pt and Au/CoFeB bilayers.

Table I. Resistivities of heavy metal and ferromagnetic layers obtained from measurements of sheet conductance. Resistivity values have been rounded up to integers and the measurement error is smaller than 1 μΩcm.

**No.**	**Sample**	**ρ_0^H (μΩcm)**	**ρ_0^F (μΩcm)**
W1	W(5)/CoFeB(t_F)/Ta(1)	185	144
W2	W(t_H)/CoFeB(5)/Ta(1)	166	144
W3	W(5)/Co(t_F)/Ta(1)	120	22
W4	W(t_H)/Co(5)/Ta(1)	120	30
P1	CoFeB(t_F)/Pt(3)	95	102
P2	CoFeB(5)/Pt(t_H)	151	161
P3	Co(t_F)/Pt(4)	51	18
P4	Co(5)/Pt(t_H)	24	57
A1	Ti(2)/Au(t_H)CoFeB(5)/Ti(1.5)	15	96
A2	Ti(2)/Au(5)/CoFeB(t_F)/Ti(1.5)	24	96

Afterwards, an in-plane magnetic field of 2 kOe, which was sufficient to achieve magnetization saturation was applied and the angular measurement of resistance as a function of azimuthal angle of magnetization was measured. Figures S3 and S4 show examples of the rotational magnetoresistance curves for investigated systems. Maximal changes of magnetoresistance ratio (MR= $(R_{max}-R_{min})/R_{min}$) as a function of the heavy metal and ferromagnet thickness were determined from in-plane angular measurements of MR curves and are collected in Figs.3-6 in the main part of this work.

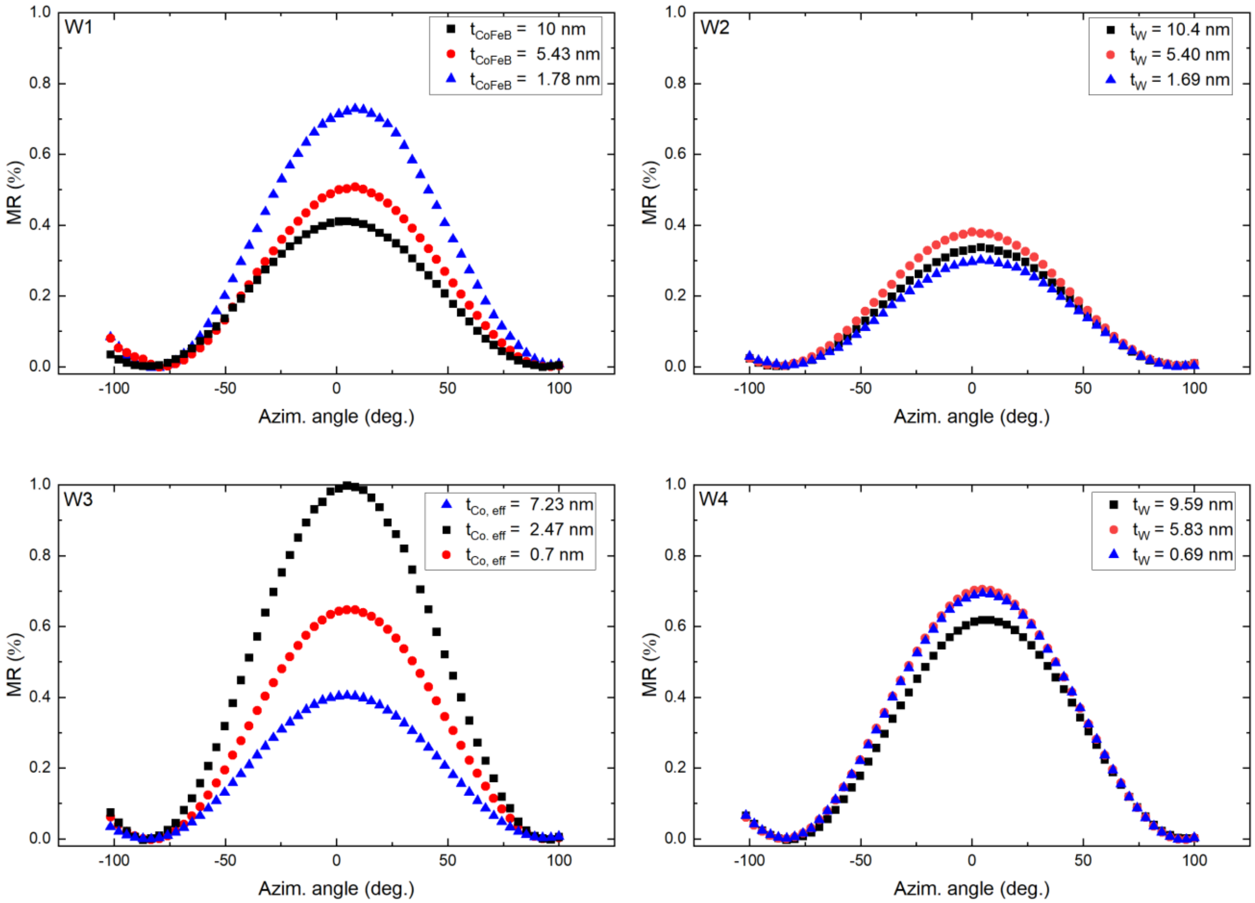

Fig. S3 The rotational magnetoresistance curves at H=2 kOe for W/CoFeB and W/Co bilayers.

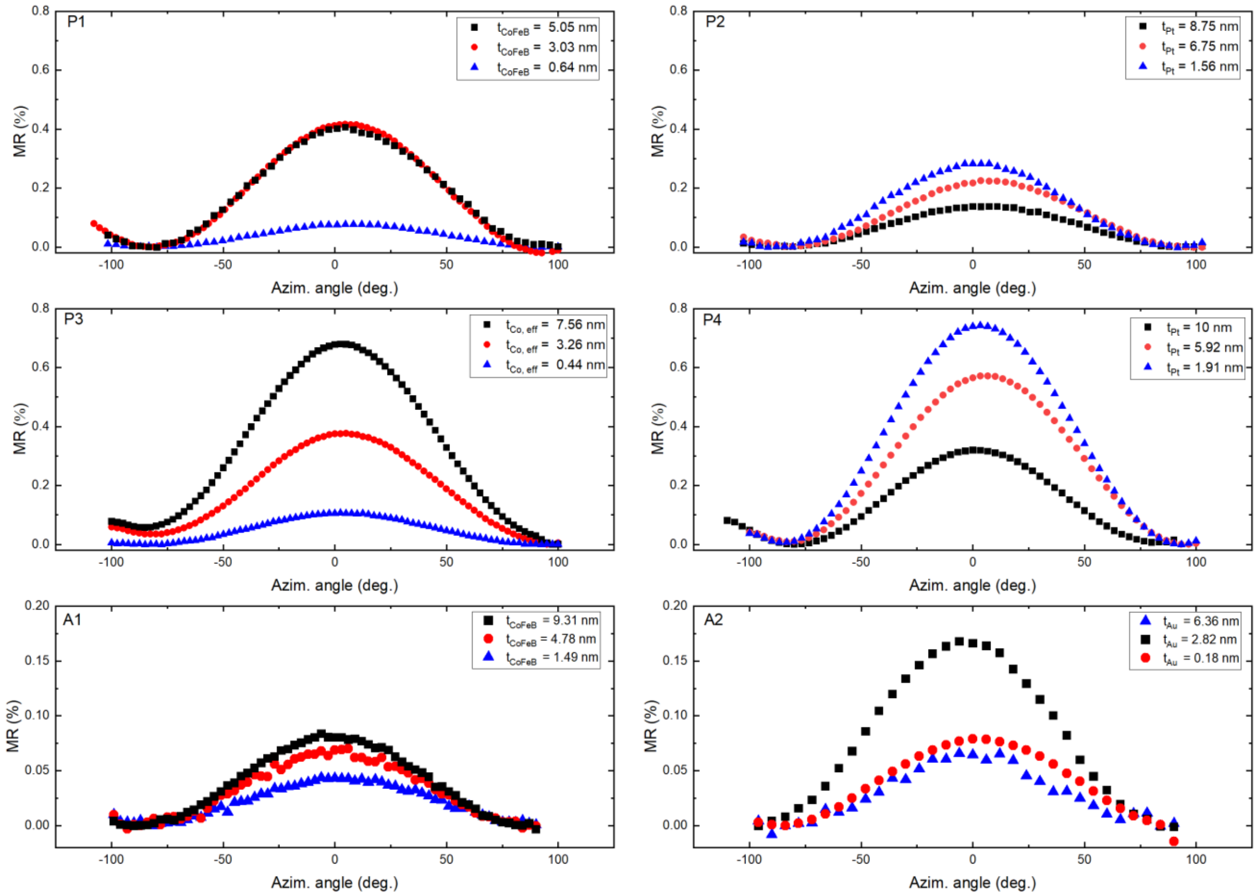

Fig. S4 The rotational magnetoresistance curves at H=2kOe for CoFeB/Pt, Co/Pt and Au/CoFeB bilayers.

References:

[S1] W. Skowroński, Ł. Karwacki, S. Ziętek, J. Kanak,S. Łazarski, K. Grochot, T. Stobiecki, P. Kuswik, F. Stobiecki, and J. Barnaś, Determination of spin Hall angle in heavy-metal/Co-Fe-B-based heterostructures with interfacial spin-orbit fields, Phys. Rev. Appl. **11**, 024039 (2019)

[S2] S. Łazarski, W. Skowroński, J. Kanak, L. Karwacki, S. Ziętek, K. Grochot, T. Stobiecki, and F. Stobiecki, Field-free spin-orbit-torque switching in Co/Pt/Co multilayer with mixed magnetic anisotropies, Phys. Rev. Appl. **12**, 014006 (2019)

[S3] M. Kawaguchi, D. Towa, Y. C. Lau, S. Takahashi, and M. Hayashi, Anomalous spin Hall magnetoresistance in Pt/Co bilayers, Appl. Phys. Lett. **112**, 202405 (2018)
